# Fostering Holistic Development with a Designed Multisport Intervention in Physical Education: A Class-Randomized Cross-Over Trial

**DOI:** 10.3390/ijerph18189871

**Published:** 2021-09-19

**Authors:** Giancarlo Condello, Emiliano Mazzoli, Ilaria Masci, Antonio De Fano, Tal Dotan Ben-Soussan, Rosalba Marchetti, Caterina Pesce

**Affiliations:** 1 Department of Medicine and Surgery, University of Parma, 43126 Parma, Italy; giancarlo.condello@gmail.com; 2 Institute for Physical Activity and Nutrition, School of Exercise and Nutrition Sciences, Faculty of Health, Deakin University, Geelong 3125, Australia; e.mazzoli@deakin.edu.au; 3 Department of Movement, Human and Health Sciences, University of Rome “Foro Italico”, 00135 Rome, Italy; ilaria.masci@gmail.com; 4 Behavioral Imaging and Neural Dynamics Center, Università degli Studi G. d’Annunzio Chieti e Pescara, 66100 Chieti, Italy; antonio.defano.gf@gmail.com; 5 Cognitive Neurophysiology Laboratory, Research Institute for Neuroscience, Education, and Didactics, Patrizio Paoletti Foundation, 06081 Assisi, Italy; research@fondazionepatriziopaoletti.org; 6 The Leslie and Susan Gonda (Goldschmied) Multidisciplinary Brain Research Center, Bar-Illan University, Ramat Gan 5290002, Israel; 7 Italian Ministry of Education, 00153 Rome, Italy; rosalba.marchetti1@gmail.com

**Keywords:** physical activity, motor competence, executive function, social emotional skills, life skills, children, enrichment, hybridization

## Abstract

Physical education (PE) is acknowledged as a relevant context for holistic child and youth development promotion. However, interventional research mostly builds on individual theories focused on specific outcome domains. This study presents a multisport enriched PE intervention that capitalizes on the intersection of different theory-based approaches to motor, cognitive and socio-emotional skills development promotion. With a cross-over design, 181 fifth graders, coming from a past class-randomized trial of enriched or traditional PE in their 1st–3rd grade, were stratified (based on their previous PE experience) and class-randomized to multisport enriched PE or control group. They completed pre-post assessments in motor and sport skills, cool (inhibition, working memory) and hot (decision making) executive functions, prosocial (empathy, cooperation) and antisocial (quick-temperedness, disruptiveness) behaviors. Children in the enriched PE group showed advantages in motor and prosocial skills after the intervention, which were linked by a mediation path, and an interactive effect of past and actual PE experience on decision making but no differential effects on other variables. The results suggest that a PE intervention designed with an integrative theory base, although not allowing disentangling the contribution of individual components to its efficacy, may help pursue benefits in motor and non-motor domains relevant to whole-child development.

## 1. Introduction

A longstanding evidence base exists on the multiple benefits of physical education (PE) for child and youth development [1]. There is consensus that PE encompasses objectives in physical-motor and socio-emotional outcome domains, even though cross-country differences exist in the prioritization of aims and objectives of current PE curricula [2].

Increasing attention to PE outcomes in the motor domain has been attracted by the flourishing of research on the development of motor competence—that is the ability to perform goal-directed movements—and its predictive value for positive trajectories of physical health [3,4] and cognitive development [5]. Curriculum-based PE shows, meta-analytically, a significant beneficial effect on overall motor competence in children and adolescents regardless of age and amount of PE time [6]. Nevertheless, a further meta-analytical comparison of interventions performed in PE and other physical activity (PA) settings [7] shows larger effect sizes (ES = 1.50) for specifically tailored motor interventions than for traditional PE classes (ES = 0.52). Also, when comparing interventions within the PE context only, in which children spent the same time in PE (i.e., same setting and dose), PE interventions specifically designed to target motor skills were more effective than typical PE [8]. This suggests that to reap the largest gains in motor skill competence, what matters is the quality of PE content and expert delivery, which is most efficacious if teachers are trained in a theoretically framed manner aimed at the acquisition of appropriate pedagogical content knowledge [9].

Expert delivery of quality PE becomes even more important to pursue objectives in the cognitive and socio-emotional domains. Indeed, the United Nations Educational, Scientific and Cultural Organization [10] indicates PE quality as a key factor to aid the development and learning of psychosocial skills. Nevertheless, a review by Opstoel et al. [11] revealed that in the last decade, only 26 studies in PE (of which only 9 interventional controlled trials) focused on personal and social development objectives grounded on different theoretical frameworks that fall under the umbrella term of ‘positive youth development’ [12].

A major framework is that of life skills, a comprehensive term used by the World Health Organization [13] to indicate an array of cognitive and socio-emotional skills that enable to deal effectively and adaptively with demands and challenges in every life domain and can be trained in educational contexts [14]. Life skills training strategies range from the creation of an implicitly favorable context and climate to the explicit discussion and practice of life skills and their transferability to other life domains [15,16]. PE and sport are considered best suited to life skills education [17]. In the PE/sport context, similar to other contexts, life skills programs are mostly targeted to adolescents attending secondary school [18], whereas PE interventions for preadolescent primary schoolers that include elements of deliberate life skills education are still rare [19].

Other theoretical approaches address personal and social skills and their education from either a cognitive perspective, especially focusing on high-level cognition relevant for health and wealth in multiple life domains (executive functions, [20]), or from a social-emotional learning perspective [21]. These perspectives have areas of intersection, since executive functions, especially ‘hot’ executive functions involved in emotionally laden decision making and social cognition [22] are linked to children’s socio–emotional development [23]. PE is recognized as a suitable platform for integrating the social-emotional learning standards into the school curriculum [24]. Main examples of instructional models for promoting social-emotional learning through PA and sport include sport education [25] and teaching personal and social responsibility [26].

Despite the predictive value of gross- and fine-motor skill competence for executive function development [27,28] and of both motor competence and executive function for socio-emotional development [23,29], the different theoretical underpinnings of interventions tailored to aid motor, executive function, or socio-emotional development constrain the creation of holistic intervention models. A way out of ‘silo-thinking’ in the design of quality PE interventions for whole-child development may be to capitalize on the intersections among motor, cognitive and socio-emotional developmental domains, as well as among theory-based approaches that have been proposed to foster domain-specific developments in the context of PE and sport [30]. Indeed, the possibility of hybridization has been proposed, meaning that students may experience differences among approaches and commonalities that facilitate their connection [31,32].

A context that may help capitalize on commonalities among theory-based interventions in PE to create an integrative approach is that of team sport games, which seems best suited to combine motor skill learning, cognitive stimulation, and life skills education. Indeed, a traditional model widely adopted in PE [33], which was originally not rooted in theory but was retrospectively added a theoretical scaffolding [34] is that of ‘teaching games for understanding’ (TGfU). It focuses on sport games, prioritizing tactical comprehension and decision-making skills. This model harmonizes with a more recent, theory-based ecological dynamics approach to skill learning in PE and sport, whose main pedagogical principles—the constraint-led approach (CLA)—focus on the interaction between learners and the environment that constrains their action, with the teacher acting as a facilitator of this interaction [35,36]. Beyond their distinctive features, TGfU and CLA have some commonalities that converge on a holistic engagement of learners physically, cognitively, and emotionally [37]. Team sport practice is also associated with higher executive function skills as early as childhood [38,39,40], suggesting a beneficial cognitive challenge of game play (‘cognitive stimulation hypothesis’, [41]). Moreover, team sport games are a privileged context for life skills program implementation in PE [17], due to the inherently high and emotionally laden social interaction. If embedded in a multisport context, team games also implicitly provide the opportunity to experience the transfer of life skills across different sport contexts [18].

Thus, the primary aim of the present study was to verify the effects of an integrative, theory-based enriched PE intervention with a multisport design, centered on team games that capitalize on commonalities among motor, cognitive and life skill-based interventions. According to the piecemeal evidence—summarized above—of benefits that can be obtained using individual intervention approaches, we hypothesized that such an integrative approach would elicit multiple gains in motor, cognitive and life skill domains.

The secondary aim was to verify, with a cross-over design, whether the expected multi-domain benefits of this integrative PE intervention performed at the end of the primary school cycle were influenced by a past experience of enrichment in PE [42]. Since the revisited model of TGfU [33,34] considers the role of cognitive skills and strategic knowledge acquired in previous experiences, we hypothesized that the cognitive skills and knowledge fostered by the past enriched PE experience would influence the effects of the actual intervention on performances that rely on them.

Our last, and exploratory, aim was to evaluate to what extent the expected benefits in the motor domain following the intervention may be linked to those in non-motor domains. Mediation evidence from the previous cognitively enriched PE intervention [42] and other similar trials of PE enrichment [43] show that gains in motor skill competence mediate gains in cognitive function. Considering this, and further evidence of associations of motor and cognitive skills with social skills [23,29], we tested whether the intervention effects on executive function and social behaviors were mediated by gains in motor competence.

## 2. Materials and Methods

The study is part of a broader longitudinal research program approved by the Ethics Committee of the “Umberto I” hospital of the First Rome University (Ref. No. 2950) and authorized by the school Committees and students’ parents, who gave written informed consent.

### 2.1. Study Design

With a cross-over design, 5th-grade primary school children coming from a past class-randomized controlled trial (RCT) of enriched or traditional PE were stratified and class-randomized to an multisport enriched PE intervention or a control group. The previous RCT involved 36 classes, comprised of 12 preschool, 12 1st–2nd grade, and 12 3rd–4th grade classes that participated in a two-year intervention (for first year intervention outcomes see [42]).

**Figure 1:**
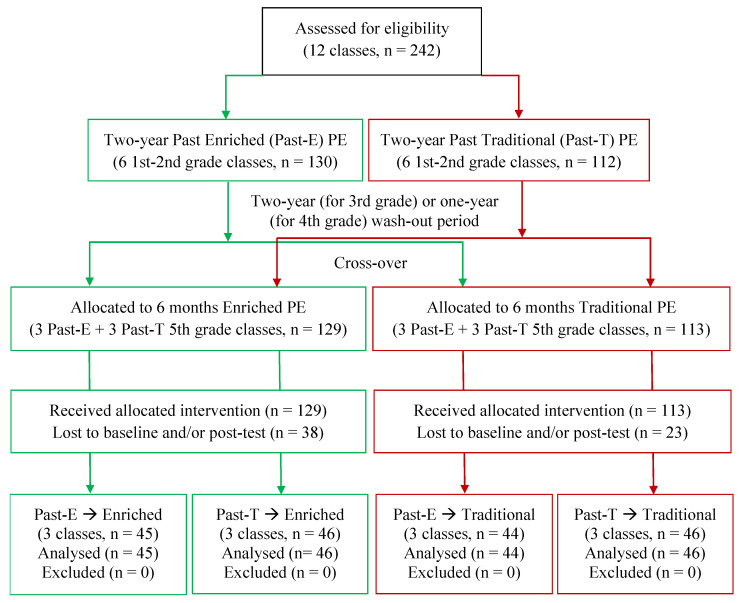
Study flow diagram for the class-randomized cross-over trial.

Only the 12 1st–2nd grade classes of the past class RCT were eligible for the present intervention. The 12 preschool and 12 3rd–4th grade classes were not eligible, because in the time frame of the first class RCT and wash-out period, they moved from preschool to primary school, or from primary to middle school, respectively, and the integrity of classes could not be maintained. The 12 eligible 1st–2nd grade classes were all recruited. They completed the past intervention phase in the 3rd–4th grade and underwent a wash-out period of two years or one year, respectively, to be involved in the present one-year trial in the 5th grade. According to a cross-over design, these classes either randomly crossed over, or maintained the same assignment in the actual intervention phase (Figure 1).

The participants were tested twice during the curricular school time: at baseline and at six months after baseline, corresponding to the end of the school year. Primary outcomes of this holistic, multi-domain intervention were motor skill competence (motor domain), ‘cool’ and ‘hot’ executive functions (cognitive domain) and prosocial, such as cooperation, empathy, and antisocial behaviors, e.g., quick-temperedness, disruptiveness (socio-emotional life skills domain). Secondary outcomes were sport-specific skills in the main sport games (soccer and basketball) included in the multisport approach to whole child development. Prior to the start of the intervention, background information on age, gender, ethnicity, body weight and height for body mass index (BMI) computation and children’s structured and unstructured PA habits were collected.

### 2.2. Participants

Two hundred and forty-two 5th-graders aged 10–11 years at baseline, belonging to twelve classes of two urban schools in the municipality of Alba (a small-size town in the Northern of Italy) participated in the study. The progress through the phases of enrolment, cross-over, intervention allocation, and final sample for data analysis is represented in Figure 1. No children failed to start the intervention after assignment or withdrew from the intervention. Loss of data (25%) was due to children’s absence on testing days pre (t1) and/or post-intervention (t2), which could not be rescheduled due to school or teacher restriction. The pattern of missingness was analyzed using the Little’s Missing Completely at Random (MCAR); it returned a non-significant result (*χ*^2^ (281, *n* = 242) = 300.86, *p* = 0.199), suggesting that data were missing completely at random. Moreover, children with missing vs. complete data did not differ in demographics. Thus, no data imputation was applied and the analyses were conducted using only the observed data after exclusion of cases with incomplete data, assuming that MCAR would reduce sample size and power, but not cause any systematic error [44]. Socio-economic status, considered sensitive information by the schools, could not be assessed. However, the schools involved in the small-sized town were not in socioeconomically deprived areas.

### 2.3. Intervention

#### 2.3.1. Duration, Setting, Blinding and Fidelity

The intervention was performed in the curricular PE time for one hour once a week, as prescribed by school regulation, and lasted for six months from November to April with a total amount of 24 intervention hours. Both the enriched and the traditional PE took place in the gym or sports court of the school with a teacher-child ratio of about 1:20 in the control classes, which was altered in the intervention classes due to the presence of the additional specialist PE teacher. However, the alteration of teacher-child ratio was minimal, as specialists were the actual deliverers and generalists mainly limited their intervention to supporting the integration of individual children with certified or non-certified health conditions. Due to of the presence of the PE specialist, teacher and children could not be blinded with respect to the assignment to generalist-led or specialist-led PE but were blinded with respect to the expected outcomes.

To ensure implementation fidelity and, at the same time, an adequate degree of adaptability, PE specialists underwent teacher training every six weeks and met regularly to align the contents of each of the four six-week teaching modules across classes and discuss teaching issues arisen in the previous module. While ensuring a consistent delivery of contents, some adaptations (i.e., game variations) were allowed. The fidelity of game variations to the module contents was estimated by means of predefined flow diagrams for task analysis, and identification of task demands in four domains: physical fitness (cardiovascular and muscular), motor coordination (functional motor control and perceptual-motor adaptation), and team-game relevant cognitive functions (with focus on ‘hot’ and ‘cool’ hot executive functions) and life skills (with focus on socio-emotional skills). During training, teachers were taught how to create nuanced game variations according to the theory-based approaches outlined in the next section. Instead, generalist teachers of the control classes were instructed to perform their ‘business as usual’. No adverse events or side effects occurred in enriched or traditional PE classes.

#### 2.3.2. Content and Delivery at the Intersection of Four Theory-Based Approaches

**Figure 2:**
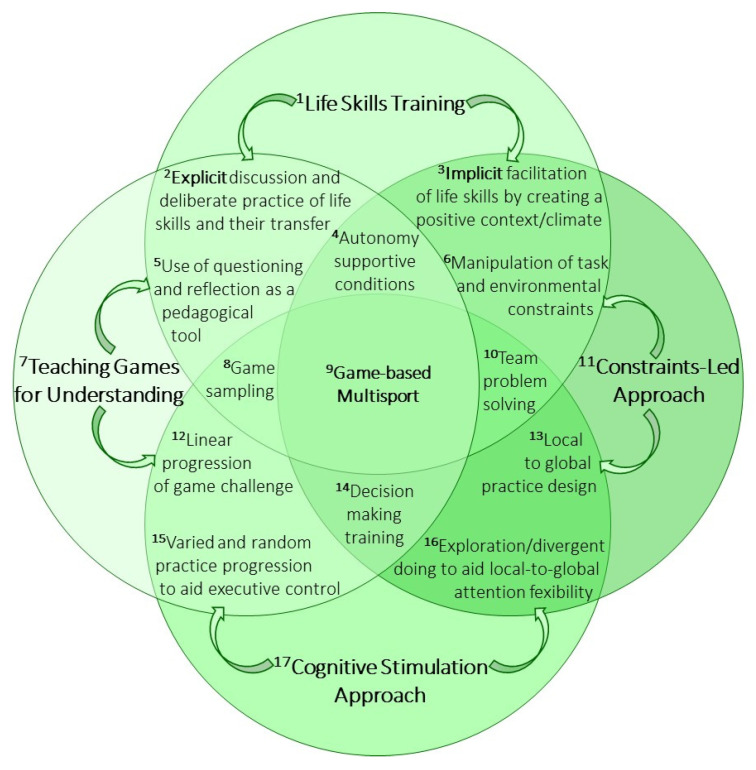
Integrative, theory-based enriched PE intervention with a multisport design centered on team games. **1**: [16,17]; **2**,**3**: [15]; **4**: [51,52]; **5**,**6**: [37]; **7**: [33,34]; **8**: [33]; **9**: [18,53]; **10**: [54]; **11**: [47,48]; **12**,**13**: [37]; **14**: [55,56]; **15**: [57]; **16**: [58]; **17**: [41,49].

To pursue holistic child development goals in multiple motor and non-motor domains, the intervention was designed in a theory-based manner, including key elements of the enriched PE intervention that preceded the cross-over trial [42,45]. However, according to the progression of objectives of current PE and school sport curricula from motor literacy to sport-related knowledge and skills transitioning from lower to higher school grade levels [46], the enriched PE program for 5th-graders involved in the present study was implemented in a multisport PE context centered on team games. Its peculiar features lie at the intersection of four main approaches/strategies of education in and through PA and sport with different theoretical underpinnings: life skills training [16,17], TGfU [33,34], CLA [47,48] and cognitive stimulation approach [41,49]. Since the operationalization of all teaching principles of the four approaches would not be feasible within one intervention, we purposely focused and capitalized on selected intersections (Figure 2).

To include principles of life skills training in our intervention design, we relied on recent reviews of sport-based youth life skills research that highlight implicit and explicit coaching practices [15,16]. Going beyond dichotomy, Bean et al. [15] propose an implicit/explicit continuum of life skills development and transfer that ranges from the most implicit strategies of structuring a facilitative sport context and generating an appropriate motivational climate to a most explicit discussion and practice of life skills and their transfer. In the present intervention, the more explicit discussion and deliberate practice of life skills intersects with the use of questioning and reflection as a pedagogical tool that characterizes TGfU [37]. On the other hand, the more implicit facilitation of life skills by creating a positive context and climate intersects with the manipulation of task and environmental constraints that characterizes the CLA [37] (Figure 2). Thus, we did not employ a formal life skills education program but included key elements of the life skills framework as goal setting, reflection on action and an appropriate motivational climate [50]. We especially focused on the creation of autonomy-supportive conditions, exploiting the unique features of the PE/sport context to satisfy students’ need for autonomy [51,52]. Autonomy support is a common feature of TGfU and CLA, as both view teachers not as instructors but as facilitators to guide discovery and promote the concept of emergence of skills through self-directed actions by learners [37].

Further commonalities between TGfU and CLA, on which the present intervention was built, include their holistic attempts to engage learners physically, cognitively and socio-emotionally and to tailor the task demands to the learner’s capabilities [37]. However, they propose different ways to deal with individual differences, which were integrated in the present intervention. In TGfU, modified games are designed in a learner-centered manner to allow for differently skilled children learning to play them with a linear progression of game challenge. In CLA, practice is centered on the learner-environment interaction [37], with task and environmental constraints being manipulated and scaled to support the emergence of local to global tendencies in individual and team game performance [35,59]. Concretely, we manipulated task objectives, players’ number and roles, play rules, space, time and equipment as proposed to foster learning in sport game contexts [56]. The last ingredient of our holistic intervention was the cognitive stimulation, for which we identified relevant intersections with TGfU and CLA (Figure 2). We developed a TGfU-based linear progression of game challenge by moving from a more constant to a more varied practice of game actions. This progression is proposed to call executive functions into play, for example to inhibit an action plan and flexibly switch to a novel one according to changing action rules [57]. On the other hand, we challenged executive control over attention by employing a local to global practice design that requires the learner to perform local-to-global attention shifts to explore and search for different pertinent solutions (i.e., divergent doing tasks; [58,60]). These approaches were integrated as complementary paths that especially foster decision making. We relied on evidence that both TGfU-based interventions focused on tactical comprehension [55] and CLA-based ‘small-sided conditioned games’ in which exploration is constrained to facilitate the roundtrip between stability and variability of individual and team actions [56] achieve significant improvements in decision making.

As a common context, we employed that of multisport centered on team games (Figure 2). We conceived the context not merely as the background of the implementation of the intervention, but rather as a sort of ‘glue’ linking all four theoretical approaches. Indeed, the team game-based multisport facilitates learning of transferable motor and life skills [18,53], capitalizing on pedagogical principles of game sampling and linear progression of modifications [33,37] that may generate a cognitively challenging experience of (dis)similarity and transferability of skills across games [18,57]. Conversely, team game-based multisport is appropriate to complement the linear progression with a curvilinear exploration-discovery path [37], which involves not only individual but also team problem solving and exploration of team synergies [54] that may jointly challenge cognitive and socio-emotional life skills.

### 2.4. Assessment Instruments and Procedures

Given the multifaceted nature of the enriched PE intervention implemented in the multisport context, we had an array of expected outcomes to be tested in motor and non-motor domains. Measurement tools were selected according to following criteria: (1) evidence of construct validity and sensitivity to PE; (2) space and time requirements appropriate to ensure feasibility in the ecological PE context. The primary outcome in the motor domain was motor competence, composed of fundamental movement skills (locomotor, object-control and stability skills; [61]) measured as a whole using the time to completion of a skill track. Secondarily, sport-specific skills in team games central to the intervention (basketball and soccer) were also tested. The primary outcome in the cognitive domain was the salient and emotionally laden, ‘hot’ decision making [22] evaluated by expert observers in team games, i.e., a real-life context in PE [62]. Emotionally neutral and decontextualized ‘cool’ executive functions (inhibition of thoughts and working memory; [22]) were also tested. The primary outcome in the socio-emotional life skills domain was prosociality (cooperation and empathy) central to team games [63]. Antisociality (quick-temperedness and disruptiveness) was also assessed [19]. Both were measured using self-ratings and peer ratings. For each assessment instrument, validity and reliability indices from previous validation studies and from the present dataset are reported in Supplementary Material [19,64,65,66,67,68,69].

#### 2.4.1. Fundamental Motor Skill Competence

The Athletic Skill Track (AST, [64]) was used. It is feasible in the school PE context, because it is time-efficient (24 children can be tested in a 50-min PE lesson) and requires few pieces of equipment, easily available in PE settings. It is validated for children aged 6–12 years with two ten-item track versions: AST-1 and AST-2. Since both tracks produced results that were individually valid, reliable and discriminant, only one (AST-1) was used for the present study. It consisted of the following locomotive, manipulative and stability skills: (1) alligator crawl, (2) bunny hops, (3) travelling jumps, (4) throwing and catching a ball, (5) kicking and stopping a ball, (6) forward roll, (7) backward roll, (8) running backwards, (9) clambering and (10) jumping. All children were shown the AST-1 course once and, as described by Hoeboer et al. [64], performed three try-out trials and two full experimental trials with a 4–5 min rest interval. The time to compete the two trials was measured with a stopwatch.

#### 2.4.2. Sport-Specific Skill Competence

Participants were individually evaluated in (1) dribbling and (2) passing skills in basketball and soccer that represented relevant component sports within the multisport approach.

- Basketball and soccer dribbling. For basketball, the participant’s task was to dribble the ball with one hand among cones placed 3 m apart (basketball; [18]). The number of cones dribbled in 30 s was recorded. Task conditions and measurement for the soccer speed dribbling were analogous.
- Basketball and soccer short pass. For basketball, the participant’s task was to hit, with a two-hand chest-throw, three circles painted on the wall different heights from the ground [18]. The number of circles struck within 30 s was recorded. Soccer pass was assessed by means of a short pass task [18]. The participant’s task was to dribble the ball up to a line from which to pass it with a side foot shot into a hockey goal. Five attempts were scored according to passing accuracy and summed to obtain a total score.

Basketball dribbling and pass evaluation outcomes were standardized and averaged into a global basketball skill score. The same standardization and averaging was performed on soccer dribbling and pass data to obtain a global soccer skill score. These composite scores were created to obtain an overall estimate of sport-specific competence for the two team games at the core of the multisport approach, rather than specific estimates of technical skills that were not a priority according to the TGfU approach [33].

#### 2.4.3. Cognitive Executive Function

Participants were evaluated in (1) ‘cool’ executive function (inhibition and working memory) by means of selected indices from the Random Number Generation (RNG) and (2) ‘hot’ executive function (decision making in a team game context) by means of the Game Performance Assessment Instrument for Invasion Games (GPAI).

- Random Number Generation task. The RNG task is a test originally validated for adults [70] and proven feasible with children aged five years and older [71]. Children were tested individually. They were presented the RNG as a game involving numbers between 1 and 10 and were asked to jumble up the numbers as much as possible at random, as if they were rolling a dice in their heads. The random number generation was paced by 70 beats with an inter-beat interval of 1.5 s. The 70-number generation sequence was preceded by an identical familiarization trial. Both the omission of a number generation in correspondence to one tone and the production of numbers lower than 1 (0) or higher than 10 (11, 12 etc.) were considered errors and discarded. Eighteen different randomness indices were computed [70] and six of them were selected, which reflect the ability to inhibit mental counting routines (turning point index -TPI-, adjacency score -Adj-, and runs score -Runs-) and the ability to update information held in working memory (redundancy score -Red)-, coupon score -Coupon-, and mean repetition gap -MeanRG-) ([66] for an extensive description). Average indices of inhibition and working memory were computed. Since, however, high levels of TPI and MeanRG, but low values of Adj, Runs, Red and Coupon reflect a good ability to suppress the habitual tendency to count forward/backward and to update information held in working memory, before averaging, all indices were z-standardized and Adj, Runs, Red and Coupon were reversed before averaging.
- Game Performance Assessment Instrument for Invasion Games. The GPAI [67] was used as a real-world proxy indicator of hot executive functions in a social and emotionally laden team game context in PE. Among seven game components observable with the multidimensional GPAI system, we assessed two that reflect the capability to make appropriate decisions for action in ball possession (Decision Making) and off-the-ball (Support). In the present study, participants’ performance during a handball game was videotaped and evaluated by two independent researchers, blind to the group assignment, on a five-point Likert scale, with higher scores indicating better decision making for action based on the proportion of observed appropriate and inappropriate actions. Decision making in ball possession involved passing actions, in which the player had to decide between several options. Judgment criteria regarded the appropriateness of choice when passing, as passing to unguarded teammates to set up a scoring opportunity. Decision making for off-the-ball support involved performing actions without ball possession, in which the player had to decide between several options for supporting ongoing action development. Judgement criteria involved the effectiveness of off-the-ball movements to receive a pass from teammates, such as freeing oneself from the opponents and proceeding to the goal. Decision making and support were merged to obtain an overall estimate of strategic decision making with or without ball possession.

#### 2.4.4. Socio-Emotional Life Skills: Prosocial and Antisocial Behaviors

The Multisource Assessment of Social Competence Scale (MASCS) designed by Junttila et al. [72] for the elementary school context was employed in its adapted version by Magotsiou et al. [69], composed of two prosocial subscales (cooperation, five items and empathy, six items reduced to five for analysis because of a cross-loading item in the present sample) and two antisocial subscales (quick-temperedness and disruptiveness, reduced from six to five items, each according to the confirmatory factor analysis by Pesce et al. [19]). Thus, each scale was composed of five items. Participants evaluated themselves and were evaluated by peers by providing responses on a five-point Likert scale anchored by 1 (I absolutely disagree) and 5 (I absolutely agree). Examples of self-evaluation items for each scale are: “I can cooperate with my classmates” (cooperation), “I am sensitive to what other pupils feel” (empathy), “I get upset very easily” (quick-temperedness), and “I am disruptive in my class” (disruptiveness). A peer-rating was obtained for each child by averaging ratings from a subset of six classmates assigned randomly at pre-test and maintained consistent at post-test, so that eventual interpersonal dyadic and rater effects influencing peer-rating were random within each class and possibly constant at pre- and post-test. We did not collect ratings by teachers, because they could not be blinded.

#### 2.4.5. Background Variables

At baseline, children’s body height and mass were measured for BMI (kg/m^2^) computation and identification of lean and overweight children based on age-referenced cut-off values of BMI. Children’s spontaneous outdoor play habits were estimated by means of the Children’s Outdoor Play assessment questionnaire ([73]; Italian validation by [42]). Parents reported the number of days their child spent at least 10 min playing in locations such as their yard at home, a friend’s or neighbor’s yard, their street or court or footpath, a park or playground in out-of-school hours on weekdays (8 items on a five-point scale) and weekend days (8 items on a six-point scale) during a typical week. Parents also filled in a questionnaire regarding their children’s actual practice (e.g., number of days/week, session duration) of after-school sports or any other structured PA training.

### 2.5. Preliminary Analyses

#### 2.5.1. Manipulation Checks

Physical exercise intensity was indirectly estimated by assessing children’s perceived exertion during a representative PE lesson in a subsample of 88 children, stratified (4–5 per class, 44 each for enriched and traditional PE) and randomly sampled. The instrument used is the stepping pictorial format of the children’s OMNI Rating of Perceived Exertion Scale (OMNI RPE), anchored by 0 (not tired at all) and 10 (very, very tired) [74]. This manipulation check was included to evaluate whether the hypothesized effect of ‘enrichment’ in PE, if found, would be coupled with a different physical exertion in specialist-led enriched PE and generalist-led traditional PE classes. A significant difference emerged (pairwise *t*-test for independent samples: *t*(86) = 5.55, *p* < 0.001), with children assigned to the enriched PE reporting, on average, ‘getting more tired’ (4.11 ± 3.10) and those assigned to the traditional PE ‘not tired at all’ or ‘a little tired’ (1.27 ± 1.39).

#### 2.5.2. Design Effect

In this class-randomized cross-over trial, children in the experimental and control groups were clustered in 12 classes belonging to two different schools. Since observations within each cluster (class) are not independent, the cluster design effect was computed. This value was used for sensitivity analysis instead of an a priori power analysis, since in our cross-over of a class-randomized trial, sample size was constrained by the recruitment of the 12 eligible classes that in the previous intervention phase were 1st and 2nd grades. These computations indicate that the actual sample size was adequate to detect effects on the primary outcome in the socio-emotional domain (see Supplementary Material) [75,76,77].

#### 2.5.3. Baseline Differences

- Baseline differences as a function of group. Pre-test values for background variables (age, BMI, outdoor play, structured PA) and for variables to be entered into the main analysis (motor skill competence, basketball skills, soccer skills, decision making, inhibition, working memory and self- and peer-rated cooperation, empathy, quick-temperedness, and disruptiveness) were submitted to *t*-tests with group (enriched PE vs. control) as a factor. Results showed significant group differences only for self-rated cooperation skills (*t*(179) = 2.72, *p* = 0.013), with children assigned to the enriched PE scoring higher than those assigned to traditional PE.
- Baseline differences as a function of past PE experience and wash-out length. We tested whether a previous experience of enriched PE in 1st–3rd grades determined differences that were maintained at the actual intervention start in the 5th grade (Past Experience effect) and whether eventual differences depended on the length of the wash-out period (Past Experience × Wash-out). To this aim, pre-test values of all motor (motor competence, basketball and soccer skills), executive function (inhibition, working memory, decision making) and socio-emotional skills (self- and peer-rated cooperation, empathy, quick-temperedness and disruptiveness) were examined using linear mixed models. Fixed effects were computed for past experience (enriched vs. traditional PE) and wash-out duration (1 vs. 2 years) and their interaction. Random effects were computed to account for clustering of children in classes. Significant results were found for decision making only: a main effect for Past Experience (*F*(1,12) = 6.42, *p* = 0.026) showed an advantage for children with past enriched PE experience compared to their traditional PE counterparts regardless of washout length (2.56 ± 1.07 vs. 2.03 ± 0.95, Cohen’s *d* = 0.53).

### 2.6. Statistical Analysis

#### 2.6.1. Primary and Secondary Hypotheses of Main and Moderated Intervention Effects

To test the hypothesis of intervention effects on overall motor skill competence, sport-specific skills, cognitive executive functions, prosocial and antisocial behaviors, we used linear mixed models. Fixed effects were computed for actual group (enriched vs. traditional PE), time (pre vs. post), past experience (enriched vs. traditional PE) and their interactions. Random effects were computed to account for clustering of children in classes. Age, gender and baseline values of outdoor play and structured PA were included as covariates. Gender was included as a covariate in consideration of the ongoing discussion on gender differences in motor skill competence [78], executive function [79] and socio-emotional skills [80]. Planned pairwise comparisons (*t*-tests) were run in the case of significant interactions. Bonferroni correction was applied to account for three comparisons (*p* < 0.016) in the post hoc analysis of two-way Actual Group × Time interactions (pre-post comparisons separately for the enriched and traditional PE group and between-groups comparison at post-test) and six comparisons (*p* < 0.008) for three-way Actual Group × Time × Past Experience interaction (same comparisons separately for each combination of Past Experience and Actual Group).

#### 2.6.2. Exploratory Hypothesis of Interrelated and Mediated Effects

- Correlation between pre-post delta values. To verify if intervention effects in the different domains were associated, bivariate correlation analyses (Pearson’s *r*) were run among delta values (Δ = [post − pre]) computed, separately for the enriched and traditional PE group, for those variables that showed differential effects of the enriched and traditional PE in the primary analysis. In the case of significant results, correlation coefficients in the two groups were tested against each other to verify if there was a significant difference in correlation.
- Mediation analysis. In the case of enriched PE effects in both motor and non-motor domains, the mediating role played by changes in motor competence was tested. To this aim, regression analyses were performed on pre-post Δ values to assess the effects of: (1) the independent variable (X: PE intervention type) on the dependent variable (Y: pre-post Δ in non-motor domain); (2) the independent variable on the mediator (M: pre-post Δ in motor domain); (3) the independent variable (X) and the mediator (M) on the dependent variable (Y). Bootstrapping was applied to empirically estimate the sampling distribution of the indirect effect and generate a bootstrap confidence interval (95% CI). This CI was used as a hypothesis test to estimate if the size of the indirect effect of the mediator was different from zero [81].

## 3. Results

### 3.1. Sample Characteristics

The final sample consisted of 181 children. There were no certified cases of neurodevelopmental and mental health conditions except for three children diagnosed with mild intellectual-relational disability or developmental learning disorder. Baseline children’s background characteristics (i.e., age, gender, ethnicity, BMI, and spontaneous and structured PA habits) and pre-post values of the variables submitted to the analysis of intervention effects are reported in Table 1.

### 3.2. Intervention Effects

#### 3.2.1. Fundamental Motor Skill Competence

**Figure 3:**
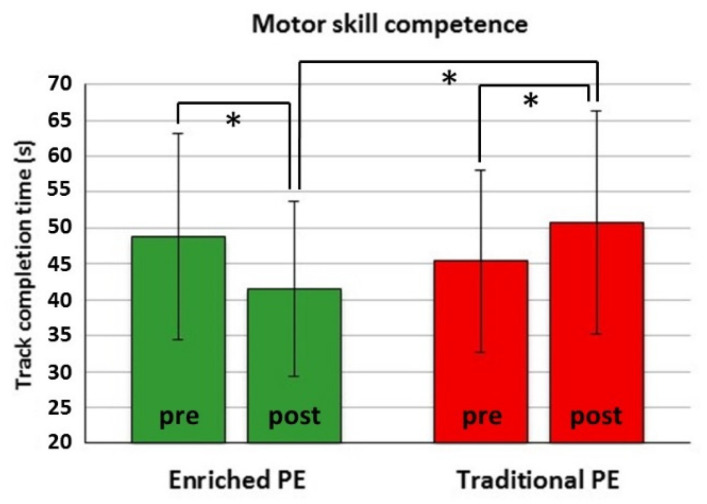
Completion time (s) of the Athletic Skill Track before (pre) and after (post) the actual enriched PE or traditional PE. * *p* < 0.016 (adjusted for three comparisons).

There was only a significant Actual Group × Time interaction (*F*(1151) = 51.60, *p* < 0.001), with ICC = 0.041. Post hoc comparisons showed a significant pre-to-post shortening of completion time in the enriched PE group (*t*(90) = 5.87, *p* < 0.001, Cohen’s *d* = 0.55), but a lengthening in the traditional PE group (*t*(89) = −5.05, *p* < 0.001, Cohen’s *d* = 0.38), leading to a significant group difference at post-test (*t*(179) = −4.47, *p* < 0.001, Cohen’s *d* = 0.66) (Figure 3).

#### 3.2.2. Sport-Specific Skill Competence

- Basketball. Only a main effect for Time emerged (*F*(1151) = 112.86, *p* < 0.001), with an overall performance increment from pre- to post-test in both groups (Table 1; Cohen’s *d* = 0.72).
- Soccer. Only a main effect for Time emerged (*F*(1151) = 5.88, *p* = 0.016), with an overall performance increment from pre- to post-test in both groups (Table 1; Cohen’s *d* = 0.14).

#### 3.2.3. Cognitive Executive Function

**Figure 4:**
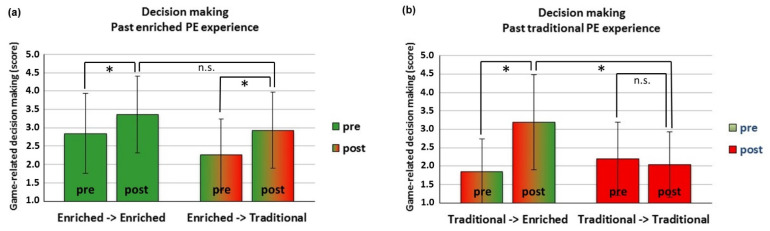
(**a**,**b**). Average value of game-related decision making and support scored with the Game Performance Assessment Instrument for Invasion Games before (pre) and after (post) the actual enriched PE or traditional PE intervention. Panel (**a**): past enriched PE experience; panel (**b**): past traditional PE. * *p* < 0.016 (adjusted for tree comparisons); n.s.: non-significant.

- ‘Cool’ executive functions. Inhibition: only a main effect for Time emerged (*F*(1151) = 29.47, *p* < 0.001), with an overall performance increment from pre- to post-test in both groups (Table 1; Cohen’s *d* = 0.43). Working memory: no significant effects emerged.
- ‘Hot’ executive functions. There were a main effect for Time (*F*(1151) = 51.96, *p* < 0.001) and significant two-way (Actual Group × Time, *F*(1151) = 21.55, *p* < 0.001) and three-way (Past Experience × Actual Group × Time, *F*(3,30) = 6.77, *p* = 0.001) interactions, with ICC = 0.23. Post hoc comparisons were performed for the highest-level interaction to identify how any differential pre-post change in the two actual PE groups was influenced by past PE experience. Children who had a past experience of enriched PE showed a pre-to-post increment in their decision-making score regardless of their actual assignment to the enriched PE (*t*(44) = −4.24, *p* < 0.001, Cohen’s *d* = 0.05) or traditional PE (*t*(43) = −4.33, *p* < 0.001, Cohen’s *d* = 0.07), reaching a non-significantly different scoring at post-test (Figure 4a). In contrast, children who had no past experience of enriched PE showed a pre-to-post gain only if they participated in the actual program of enriched PE (*t*(45)= −7.15, *p* < 0.001, Cohen’s *d* = 0.12) but no gain if they had neither a past, nor an actual experience of enriched PE (*p* < 0.305). This differential result led to a different scoring of the two groups at post-test (*t*(90) = −4.95, *p* < 0.001, Cohen’s *d* = 0.10) (Figure 4b).

#### 3.2.4. Prosocial and Antisocial Behavior

**Figure 5:**
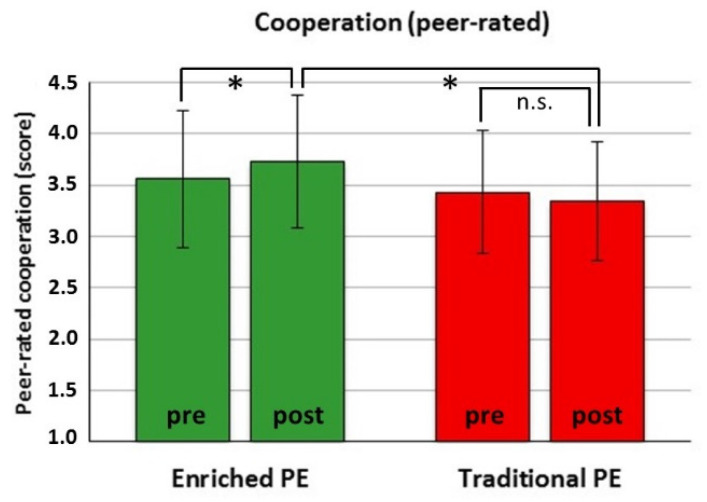
Peer-rated cooperation scored with the Multisource Assessment of Social Competence Scale before (pre) and after (post) the actual enriched PE intervention or the traditional PE. * *p* < 0.016 (adjusted for three comparisons); n.s.: non-significant.

**Figure 6:**
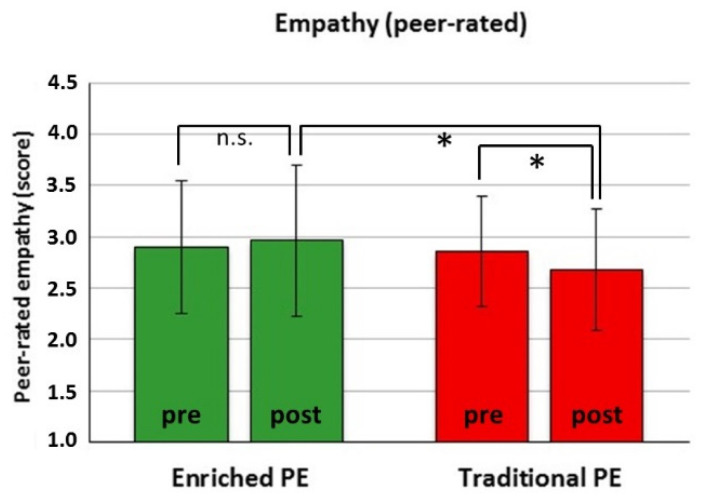
Peer-rated empathy scored with the Multisource Assessment of Social Competence Scale before (pre) and after (post) the actual enriched PE intervention or the traditional PE. * *p* < 0.016 (adjusted for three comparisons); n.s.: non-significant.

- Prosocial behavior. For self-rated cooperation, there was no significant effect. For self-rated empathy, there was only a main effect for Time (*F*(1151) = 5.08, *p* = 0.026), with an overall score increment from pre- to post-test in both groups (Table 1; Cohen’s *d* = 0.15). For peer-rated cooperation and empathy, there was a significant Actual Group × Time interaction (cooperation: *F*(1151) = 7.73, *p* = 0.006; empathy: *F*(1151) = 6.72, *p* = 0.010), with ICC = 0.15 and 0.08, respectively. For peer-rated cooperation, also a three-way interaction (Past Experience × Actual Group × Time, *F*(3,36) = 5.53, *p* = 0.003) emerged. However, post hoc analysis did not show differential simple effects for the Actual Group × Time interaction as a function of Past Experience. Post hoc analysis of the two-way interactions showed a significant pre-to-post increase in peer-rated cooperation in the enriched PE group (*t*(90) = −3.21, *p* = 0.002, Cohen’s *d* = 0.26) but a non-significant decrement in the traditional PE group (*p* = 0.166), leading to a large, though marginally significant (for adjusted *p* < 0.016) group difference at post-test (*t*(179) = 2.38, *p* = 0.018, Cohen’s *d* = 0.62) (Figure 5). Instead, peer-rated empathy showed no significant pre-to-post increase in the enriched PE group (*p* = 0.177) but a decrement in the traditional PE group (*t*(89) = −2.95, *p* = 0.004, Cohen’s *d* = 0.33), leading to a significant group difference at post-test (*t*(179) = 2.92, *p* = 0.004, Cohen’s *d* = 0.43) (Figure 6).
- Antisocial behavior. There was only a main effect for Time for self-rated quick-temperedness (*F*(1151) = 4.83, *p* = 0.029) and disruptiveness (*F*(1151) = 7.43, *p* = 0.007), with an overall score decrement from pre- to post-test in both groups (Table 1; Cohen’s *d* = 0.19, and 0.23, respectively). For peer-rated quick-temperedness and disruptiveness, no significant effects emerged.

### 3.3. Correlation and Mediation Effects

#### 3.3.1. Correlation between Pre-Post Delta Values

Bivariate correlations were computed, separately for the enriched and the traditional PE groups, between pre-post Δ values of those variables, which showed differential intervention effects in the primary analysis: motor skill competence, decision making, peer-rated cooperation and empathy. There was only one weak but significant negative correlation between Δ motor competence and Δ peer-rated cooperation in the enriched PE group (*r* = −0.22, *p* = 0.019), indicating that in the enriched PE group, shorter times to completion of the motor skill track were linked to higher peer-ratings of cooperation. Instead, the traditional PE group did not show this association (*r* = −0.09, *p* = 0.193). However, this weak difference in correlation between groups did not reach significance (*z* = −0.87, *p* = 0.193).

#### 3.3.2. Mediation of Gains in Non-Motor Domains by Gains in Motor Competence

**Figure 7:**
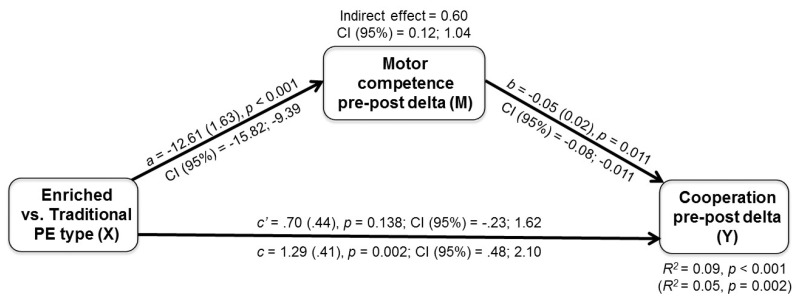
Mediation model: effect of PE intervention type (‘X’: enriched vs. traditional) on pre-to-post change in peer-rated cooperation skill (‘Y’) mediated by pre-to-post change in motor skill competence (‘M’). a, b, c: regression coefficients with (SE), p and CI (95%) values. c: total effect; c’: direct effect after accounting for the mediator. *R*^2^ values with/(without mediator) and bootstrap CI (95%) for indirect effects are also reported.

The pre-post Δ motor competence was tested as a mediator of the enriched PE effects found on decision making and peer-rated cooperation and empathy. Consistently with the correlational analysis, the pre-post Δ motor competence was found to mediate the beneficial effect of the enriched PE intervention on peer-rated cooperation only. As indicated by the bootstrapping output (Figure 7), the 95% CI of bootstrap estimates of the indirect effect did not include the zero value. The same model applied to pre-post Δ decision making and peer-rated empathy did not yield any significant mediated path.

## 4. Discussion

This study aimed to verify the efficacy of an integrative, theory-based enriched PE intervention with a multisport design centered on team games to foster child development in multiple domains (primary hypothesis); it also tested whether a past experience of enrichment in PE at the beginning of the primary school cycle could influence actual intervention effects at its end (secondary hypothesis) and whether eventual gains in motor competence mediate intervention effects in non-motor domains (exploratory hypothesis).

The primary hypothesis found confirmation for motor and prosocial life skills: the results showed diverging trends of change between the enriched and traditional PE group, which led to better motor competence, cooperation and empathy in children who participated in the enriched multisport intervention as compared to those involved in traditional PE. Instead, ‘cool’ executive functions and antisocial behaviors were unaffected by PE enrichment. In relation to the secondary hypothesis, no interaction between past and actual experience of PE enrichment was found except for ‘hot’ executive function, showing a beneficial effect of past PE enrichment and a compensatory effect of the actual intervention in the absence of a past experience of PE enrichment. The exploratory hypothesis found partial confirmation: only the gain observed in cooperation skill was associated with and mediated by the increment in motor competence, whereas the gains in empathy and decision making of the enriched PE group were unrelated to the improvement in motor competence.

The amelioration of overall motor skill competence in the enriched PE group and its worsening in the traditional PE group over the intervention time (Figure 3) confirms that at the threshold to adolescence, designed specialist-led interventions have the potential to promote motor skill competence [7,8] and even prevent its deterioration. The relevance of interventions that aid motor competence development in preadolescence is especially evident when considering the predictive role of overall motor competence in childhood for later PA levels [4,82,83] and, conversely, the worldwide high rates of insufficient PA in adolescence [84]. Reviews of interventions targeted to motor competence [8,85] called for attention to the theoretical or pedagogical approaches to gain a better understanding of the features of efficacious interventions. They highlighted some key elements such as autonomy-supportive motivational climate and exploration [8], as well as a face-to-face delivery by experts and group implementation settings [85]. These elements were also featured in our intervention, and were targeted to aid not only motor competence, but also non-motor (cognitive and socio-emotional) skills (Figure 2).

Indeed, the autonomy-supportive learning conditions were purposely designed to foster life skills and have likely contributed to the beneficial effects on cooperation and empathy (Figure 5 and Figure 6), even though their relative contribution cannot be disentangled from the role played by other characteristics of our integrative approach. Both theoretical [50] and empirical works [51,52] suggest that deliberately tailored teacher support and a contextual climate that satisfies student’s need for autonomy are predictive of life skills as team work, social and emotional skills. However, those works are grounded on self-determination theory [86] that is beyond the theoretical boundaries, scope and assessments of this integrative intervention. Rather, the selective beneficial effects of our intervention on prosocial life skills were probably due to the CLA approach to team problem solving [54] and the TGfU approach to team game sampling [33].

The opportunity to sample sport games and experience the action challenges posed by different sports has likely contributed to the improvement in overall motor competence, in line with gains obtained in previous studies that adopted a multisport approach with 5th-graders as in the present study [53,87]. However, the present intervention did not merely involve the sampling of sports in a sequential fashion, but integrated a multiplicity of actions and technical skills from different sports into modified team games. This integration, which was the context for the modulation of task and environmental constraints (CLA) and training of tactical understanding (TGfU) is likely responsible for the lack of intervention effects on sport-specific skills. According to a common critique of TGfU and CLA, it may take longer for successful learning of specific sport skills to be seen [37]. Moreover, our results are hardly comparable with evidence from CLA interventions to develop interceptive sport skills [88], because they usually focus on learning a single skill and the few studies conducted with preadolescent children [89,90] examined the effects of single-session training of an individual interceptive sport.

We did not follow the properly criticized interpretation of TGfU as a cognition-to-technique approach as opposed to the motor behavioristic technique-to-cognition approach. Instead, we applied a cognitive psychology perspective framework to TGfU [33] that capitalizes on the cognitive demands of skill acquisition to foster executive function development [57,91]. In contrast to our expectation, however, we found that our team sport-based, enriched PE did not benefit ‘cool’ executive functions (inhibition, working memory) but only ‘hot’ executive functions (decision making). The lack of effects on working memory was expected both because children’s working memory seems more sensitive to the manipulation of PA quantity than PA quality [92] and because it showed no improvement already in the previous PE enrichment phase before the cross-over [42]. Instead, the lack of effects on inhibition is in contrast to meta-analytical syntheses suggesting that qualitatively enriched PA interventions focused on motor skills and cognitive engagement may foster children’s inhibition [93,94]. One reason may be a low sensitivity to PA of the specific type of inhibition assessed in the present study. Inhibitory control is multifaceted and has been mostly assessed, in exercise and cognition research, as interference control or response inhibition [95] but only rarely as inhibition of routine thoughts assessed in the present study. This latter facet of inhibition was found sensitive to PE enrichment in the original sample of the present study at early and middle childhood age [42] but not in older children [19]. Alternatively, the cognitive stimulation may have been not sufficient, being only one of the multiple foci of this integrative intervention, or not sufficiently specific, considering that executive functions exhibit narrow transfer and gains in a trained function do not transfer to another one [96]. Indeed, our intervention focused on cognitive challenge generated by multisport games. While cross-sectional evidence shows that the practice of team sports is consistently associated with better inhibitory performance [39,40,41], interventional research mostly failed to find benefits of cognitively enriched sport game interventions for pure measures of inhibition [97,98,99].

The only executive function that benefited from PE enrichment is a ‘hot’ executive function the TGfU and the CLA convergence on: game-related decision making (Figure 2). The convergence is due to the fact that ‘hot’ executive functions integrate the rational conceptualization of executive functions, attuned to the cognitive TGfU perspective [33], with the consideration of bottom-up emotional processes in cognitive control that are better attuned to the CLA perspective [35]. The positive effect of our intervention on decision-making skill is supported by evidence of efficacy of both TGfU-based training of tactical comprehension [55] and CLA-based manipulation of game features and risk taking [56] that is a distinguishing feature of decision making in ‘hot’ executive function research [22]. Moreover, the assessment of decision making in the emotionally salient team game setting seems best suited to detect a decision-making skill generalizable to other life domains, as it was found predictive of self-regulation in everyday life [19]. Thus, the ability of enriched PE to improve game-based decision making at the end of preadolescence has relevant implications, because adolescents may exhibit lowered ‘hot’ executive functions and enter a period of heightened risk-taking propensity [100]. Moreover, decision making seems a key factor linked to students’ academic performance [101].

Our results showed both a longer- and a shorter-term benefit for decision making. The first is the benefit of the past experience of PE enrichment that is still maintained three years later. An early experience of PE enrichment at the beginning of the primary school cycle seems to be a longer-term investment. Indeed, children who could make this early investment reached the 5th grade with a higher level of decision-making skill and further improved over time regardless of being involved in the further phase of enriched team game-based or traditional PE (Figure 4a). Probably, the skills acquired in the first PE enrichment phase and maintained over years allowed children to perceive and exploit the affordances offered by other players and by the play environment to further learn decision making. The shorter-term benefit is compensatory: children who, having no past experience of enriched PE, started the actual enriched PE intervention with a lower baseline level of decision-making skill caught up, showing the largest improvement over the intervention time. Instead, the only children who did not improve at all where those who never experienced PE enrichment along the primary school cycle (Figure 4b). This is consistent with a study showing that a TGfU intervention elicited the largest and significant gains in decision making in children with a low baseline level of tactical decision-making skills [102].

The gains in decision making and motor competence were not associated, nor this latter mediated the intervention effects on the first. This suggests that what we found is an improvement in true decision-making skill rather than a spurious outcome affected by gains in motor skills with the ball, as also shown in a previous study [19]. Instead, an interesting, though weak association emerged between cooperation and motor competence gains, with the latter partially mediating the intervention effects on the first (Figure 7). In the broadest context, this result is in line with embodied theories of social competence [103] and with the hypothesis that a holistic approach to fostering the development in motor and non-motor domains may capitalize on their interrelatedness [29]. The expected mediation of gains in cognitive function by gains in motor competence [42,43] was, instead, not found and deserves future attention with a specific focus on those executive functions that may better match the cognitive demands of team sport games.

This study is not without limitations. Firstly, the two-group design with one group assigned to the intervention with an integrated theoretical background and the other assigned to ‘business as usual’ does not allow to unequivocally attribute the different intervention outcomes to any of its individual features, or to their additive/interactive effects. Secondly, the impossibility to blind teachers and children might have generated a Hawthorne effect that is the tendency towards higher engagement of those involved in the experimental PE enrichment. However, this limitation is attenuated by the fact that both teachers and children were unaware of the expected outcomes. A further limitation regards the non-generalizability of findings to students with socio-economic disadvantage, since all the participating schools were located in non-deprived areas. Moreover, to adhere to a curricular shift in PE focus toward sport-specific skills along the primary school cycle, the PE enrichment after the cross-over for 5th-graders was embedded in a team sport-based multisport context. This multisport context, along with the older age of the children provided the conditions to add a stronger focus on life skills training, and required the use of new assessments valid for the children’s age and consistent with the actual PE content. Finally, there are at least two side effects of the ecological validity of this intervention. The first one is the PE frequency, constrained to once a week by school regulation, which may be one of the causes of the lack of sustained impact, from the past to the actual intervention phase, on motor competence otherwise observed for school-based interventions grounded on sound theory [104]. The second one is the loss of data due to school constrains on additional testing sessions for students who were absent on testing days. Even though missing value analysis suggested missing data being completely random, the relatively high percentage of missings calls for caution in drawing conclusions. However, the potential impact of excluded missing data on the results is limited by the fact that the absence on testing days was unrelated to the intervention, as no children withdrew after group assignment.

## 5. Conclusions

PE intervention designed with an integrative theory base may help pursue benefits in motor and non-motor domains relevant to whole-child development. Beyond the limitations discussed above, this study also has strengths and novelties. It has the value of being theory-grounded while, at the same time, avoiding a narrow focus on one individual theory that often limits the development of holistic interventions with multiple foci attuned to the different goals of quality PE. The study outcomes support the advantage of hybrid over individual pedagogical models, confirming that the former can promote outcomes in many different domains, overcoming the boundaries of single theoretical approaches [31,32]. We identified and capitalized on key intersections among relevant theories that encompass motor, cognitive and socio-emotional outcomes of PE. The present study is an attempt to respond to the call for a more holistic approach to PE grounded in a broader conception, because “PE is much broader than just PA, and we harm the future potential of our field if we adopt a narrow agenda.” [105] (p. 144). Our results are encouraging and show the potential of a holistic model grounded in the intersection of multiple theories to elicit gains in motor and non-motor domains that are partly interconnected. Conversely, the lack of effects in specific outcome domains call for adaptation and refinement and for further research that operationalizes a holistic approach to quality PE by means of integrative theory-based programs.

## Figures and Tables

**Table 1:** Demographics, baseline levels of spontaneous outdoor play and structured sports training, and baseline (t1) and post-intervention (t2) values of motor skill competence, sport skills (basketball and soccer), cool executive functions (inhibition, working memory), hot executive functions (decision making in ball game), self-rated and peer-rated prosocial behavior (cooperation, empathy) and antisocial behavior (quick-temperedness/disruptiveness) of 10–11 year-old children assigned to the multisport enriched physical education (PE) intervention or traditional PE.

Variables	Enriched PE	Traditional PE
*n*	91	90
Gender (*n* females/*n* males)	45/46	45/45
Age (years ± SD)—Baseline	10.73 (±0.32)	10.72 (±0.31)
Immigrant background (%) (East-European, North-African, Hispanic)	18%	13%
Body Mass Index (BMI)	17.76 (±2.48)	18.09 (±3.14)
Lean (%)	81%	80%
Overweight (%)	19%	20%
Spontaneous outdoor play (score)—Baseline	36.60 (±12.78)	36.22 (±13.13)
Structured sports training (min/week)—Baseline	183 (±189)	204 (±208)
Motor skill competence (execution time) #*		
Pre	48.79 (±14.26)	45.43 (±12.67)
Post	41.52 (±12.16)	50.77 (±15.48)
Sport skills: Basketball (std avg correct pass & dribbling) *		
Pre	−0.21 (±0.75)	−0.27 (±0.65)
Post	0.22 (±0.85)	0.26 (±0.92)
Sport skills: Soccer (std avg correct pass & dribbling) *		
Pre	−0.05 (±0.82)	−0.08 (±0.82)
Post	0.13 (±0.85)	−0.01 (±0.87)
Cool executive function: Inhibition (std score) *		
Pre	−0.13 (±0.76)	−0.20 (±0.99)
Post	0.08 (±0.72)	0.25 (±0.61)
Cool executive function: Working memory (std score)		
Pre	−0.02 (±0.73)	−0.01 (±0.79)
Post	0.03 (±0.68)	−0.03 (±0.67)
Hot executive function: Decision making (score) *; #*; #*§		
Pre	2.35 (±1.10)	2.23 (±0.98)
Post	3.28 (±1.17)	2.48 (±1.05)
Prosocial behavior: Cooperation (self-rating)		
Pre **#**	4.00 (±0.80)	3.66 (±1.00)
Post	4.07 (±0.69)	3.76 (±0.82)
Prosocial behavior: Cooperation (peer-rating) #*		
Pre	3.56 (±0.66)	3.43 (±0.60)
Post	3.73 (±0.64)	3.35 (±0.57)
Prosocial behavior: Empathy (self-rating) *		
Pre	3.28 (±0.89)	3.08 (±0.89)
Post	3.38 (±0.85)	3.25 (±0.84)
Prosocial behavior: Empathy (peer-rating) #*		
Pre	2.90 (±0.65)	2.86 (±0.54)
Post	2.96 (±0.74)	2.67 (±0.59)
Antisocial behavior: Quick-temperedness (self-rating) *		
Pre	2.11 (±0.85)	2.14 (±0.76)
Post	2.00 (±0.85)	1.94 (±0.65)
Antisocial behavior: Quick-temperedness (peer-rating)		
Pre	2.16 (±0.75)	2.16 (±0.86)
Post	2.10 (±0.83)	2.07 (±0.79)
Antisocial behavior: Disruptiveness (self-rating) *		
Pre	1.66 (±0.64)	1.75 (±0.69)
Post	1.57 (±0.56)	1.56 (±0.51)
Antisocial behavior: Disruptiveness (peer-rating)		
Pre	1.91 (±0.67)	1.85 (±0.73)
Post	1.84 (±0.73)	1.88 (±0.74)

Note. Statistically significant effects (*p* < 0.05) are marked as follows: **#** Group at baseline (see Section 2.5.3); * Time; #* Group × Time; #*§ Group × Time × Past Experience (see Section 2.6.1 and Section 3.1).

## Data Availability

The data presented in this study are available on request from the corresponding author. The data are not publicly available due to restrictions (i.e., national privacy legislation and inclusion, in the informed consent signed by parents/guardians, only of permission for communication of the data in aggregated form).

## Supplementary Materials

The following are available online at https://www.mdpi.com/article/10.3390/ijerph18189871/s1, The validity and reliability of assessment instruments and the design effect are available as supplementary materials.
