# Supplementary material for: Fostering Holistic Development with a Designed Multisport Intervention in Physical Education: A Class-Randomized Cross-Over Trial
Source: Int J Environ Res Public Health. 2021 Sep 19;18(18):9871. doi: 10.3390/ijerph18189871 (PMC8467800; doi:10.3390/ijerph18189871)
Supplement: Supplementary file 1 [file ijerph-18-09871-s001.zip › ijerph-1337285-supplementary.pdf]

Validity and reliability of assessment instruments

- **Athletic Skill Track (AST)**. In the original validation study [64], concurrent validity, as assessed by correlating the AST-1 with the overall motor quotient of the Körperkoordinationstest für Kinder (KTK) was moderate for both females and males (r = - 0.501 and - 0.533, respectively; overall = - 0.474). Test-retest reliability between the first and second trial, was high both in the validation study (intraclass correlation coefficient [ICC] = 0.875) and in the present study (ICC = 0.915).
- **Random Number Generation (RNG) Task**. Based on the factorial structure identified by Miyake et al. [65], three indices of the RNG are reported to draw on inhibitory control and three on working memory updating (standardized regression coefficients from structured equation modelling = 0.39 and 0.33, respectively; [65]). These two three-factorial components have acceptable to high internal consistency (ICC > 0.86 and > 0.82, respectively; [66]).
- **Game Performance Assessment Instrument (GPAI) for Invasion Games**. In the first validation studies [67], both test-retest reliability (ICC) and inter-observer agreement were satisfactory ($r > 0.80$) for all game components, as well as construct validity (i.e., 66% consistency between GPAI scores and teachers' rankings of game play ability). The observed game components (decision making and support) have construct similarity [68].
- **Multisource Assessment of Social Competence Scale (MASCS)**. Psychometric properties (as reported by Magotsiou et al. [69] for all scales and by Pesce et al., [19] for the antisocial scales) show strong internal consistency (Cronbach's alpha > 0.80) and test-retest reliability for each scale (Cronbach's alpha > 0.80), as well as confirmation of an acceptable fit of the bifactorial structure of the two scales of prosocial and antisocial behavior. In the present sample, internal consistency ranged between acceptable and strong for self-rated and peer-rated scales (Cronbach's alpha = 0.69–0.79 and 0.86–0.96, respectively).

Design effect

The cluster design effect computation takes into account that the variance of the mean computed from a clustered sample is larger by a factor of [1 + (n – 1) × ICC], and, to take into account differences in cluster size (i.e., number of children tested in each class):

$$\text{Cluster design effect (clusters of different size)} = \{1 + [(\text{CV}^2 + 1) \times \text{n} - 1] \times \text{ICC}\}$$

where n = number of children within each cluster, CV = coefficient of variation for n and ICC = intraclass correlation coefficient ([$\sigma^2_{\text{between-cluster}} / (\sigma^2_{\text{between-cluster}} + \sigma^2_{\text{within-cluster}})$]; [75]).

With a mean cluster $n = 15.08$, a CV = 0.27 and an assumed ICC for the primary outcome in the socio-emotional domain = 0.15 [76], the estimated design effect was 3.28. This design effect value is usually used as a multiplier of the sample size determined with a priori power analysis. Since in our cross-over of a class-randomized trial, sample size was constrained by the recruitment of the twelve classes that in the previous intervention phase were 1st and 2nd grade classes, rather than an a priori power analysis for computing sample size we performed a sensitivity analysis for computing required effect size given alpha, power and sample size was performed. To this aim, the design effect value was used as a divisor of the actual sample size to obtain an estimate of sample size (181 / 3.28 = 55) to compute, for $\alpha = 0.05$ e $\beta\text{-}1 = 0.80$, the minimal detectable effect size (ES = 0.23). This value is in line with the effect size reported in the literature pertaining school-based interventions to foster prosocial socio-emotional skills [77], primary outcome in the life skills domain of the present holistic intervention. Thus, the actual sample size was deemed adequate to detect effects on the primary outcome in the socio-emotional domain.

References

19. Pesce, C.; Lakes, K.D.; Stodden, D.F.; Marchetti, R. Fostering Self-Control Development With a Designed Intervention in Physical Education: A Two-Year Class-Randomized Trial. *Child Dev.* **2021**, *92*, 937–958, doi: 10.1111/cdev.13445
64. Hoeboer, J.; De Vries, S.; Krijger-Hombergen, M.; Wormhoudt, R.; Drent, A.; Krabben, K.; Savelsbergh, G. Validity of an Athletic Skills Track among 6- to 12-Year-Old Children. *J. Sports Sci.* **2016**, *34*, 2095–2105, doi:10.1080/02640414.2016.1151920.
65. Miyake, A.; Friedman, N.P.; Emerson, M.J.; Witzki, A.H.; Howerter, A.; Wager, T.D. The Unity and Diversity of Executive Functions and Their Contributions to Complex "Frontal Lobe" Tasks: A Latent Variable Analysis. *Cogn. Psychol.* **2000**, *41*, 49–100, doi:10.1006/cogp.1999.0734
66. Audiffren, M.; Tomporowski, P.D.; Zagrodnik, J. Acute aerobic exercise and information processing: Modulation of executive control in a Random Number Generation task. *Acta Psychol.* **2009**, *132*, 85–95, https://doi.org/10.1016/j.actpsy.2009.06.008.
67. Oslin, J.L.; Mitchell, S.A.; Griffin, L.L. The Game Performance Assessment Instrument (GPAI): Development and Preliminary Validation. *J. Teach. Phys. Educ.* **1998**, *17*, 231–243, https://doi.org/10.1123/jtpe.17.2.231.
68. Memmert, D.; Harvey, S.; The Game Performance Assessment Instrument (GPAI): Some Concerns and Solutions for Further Development. *J. Teach. Phys. Educ.* **2008**, *27*, 220–240. doi:10.1123/jtpe.27.2.220
69. Magotsiou, E.; Goudas, M.; Hasandra, M. Validity and Reliability of the Greek Version of the Multisource Assessment of Social Competence Scale. *Percept. Mot. Ski.* **2006**, *103*, 667–675, https://doi.org/10.2466/pms.103.3.667-675.
75. Hedges, L. V; Hedberg, E.C. Intraclass Correlation Values for Planning Group-Randomized Trials in Education. *Educ. Eval. Policy Anal.* **2007**, *29*, 60–87, doi:10.3102/0162373707299706.
76. Björklund, K.; Liski, A.; Samposalo, H.; Lindblom, J.; Hella, J.; Huhtinen, H.; Ojala, T.; Alasuvanto, P.; Koskinen, H.-L.; Kiviruusu, O.; et al. "Together at School" - a School-Based Intervention Program to Promote Socio-Emotional Skills and Mental Health in Children: Study Protocol for a Cluster Randomized Controlled Trial. *BMC Public Health* **2014**, *14*, 1042, doi:10.1186/1471-2458-14-1042.
77. Durlak, J.A.; Weissberg, R.P.; Dymnicki, A.B.; Taylor, R.D.; Schellinger, K.B. The Impact of Enhancing Students' Social and Emotional Learning: A Meta-Analysis of School-Based Universal Interventions. *Child Dev.* **2011**, *82*, 405–432, doi:10.1111/j.1467-8624.2010.01564.x.
